# The Immunomodulatory Effects of Macrolides—A Systematic Review of the Underlying Mechanisms

**DOI:** 10.3389/fimmu.2018.00302

**Published:** 2018-03-13

**Authors:** Petra Zimmermann, Victoria C. Ziesenitz, Nigel Curtis, Nicole Ritz

**Affiliations:** ^1^Department of Paediatrics, The University of Melbourne, Parkville, VIC, Australia; ^2^Infectious Diseases & Microbiology Research Group, Murdoch Children’s Research Institute, Parkville, VIC, Australia; ^3^Infectious Diseases Unit, The Royal Children’s Hospital Melbourne, Parkville, VIC, Australia; ^4^Infectious Diseases Unit, University of Basel Children’s Hospital, Basel, Switzerland; ^5^Paediatric Pharmacology, University of Basel Children’s Hospital, Basel, Switzerland

**Keywords:** azalides, azithromycin, clarithromycin, erythromycin, immunolides, roxithromycin

## Abstract

**Background:**

The mechanisms underlying the non-antimicrobial immunomodulatory properties of macrolides are not well understood.

**Objectives:**

To systematically review the evidence for the immunomodulatory properties of macrolides in humans and to describe the underlying mechanism and extent of their influence on the innate and adaptive immune system.

**Methods:**

A systematic literature search was done in MEDLINE using the OVID interface from 1946 to December 2016 according to the preferred reporting items for systematic reviews and meta-analysis (PRISMA). Original articles investigating the influence of four macrolides (azithromycin, clarithromycin, erythromycin, and roxithromycin) on immunological markers in humans were included.

**Results:**

We identified 22 randomized, controlled trials, 16 prospective cohort studies, and 8 case–control studies investigating 47 different immunological markers (186 measurements) in 1,834 participants. The most frequently reported outcomes were a decrease in the number of neutrophils, and the concentrations of neutrophil elastase, interleukin (IL)-8, IL-6, IL-1beta, tumor necrosis factor (TNF)-alpha, eosinophilic cationic protein, and matrix metalloproteinase 9. Inhibition of neutrophil function was reported more frequently than eosinophil function. A decrease in T helper (Th) 2 cells cytokines (IL-4, IL-5, IL-6) was reported more frequently than a decrease in Th1 cytokines (IL-2, INF-gamma).

**Conclusion:**

Macrolides influence a broad range of immunological mechanisms resulting in immunomodulatory effects. To optimize the treatment of chronic inflammatory diseases by macrolides, further studies are necessary, particularly comparing different macrolides and dose effect relationships.

## Background

Macrolides are mainly used as antibiotics to treat respiratory, skin and soft tissue, and urogenital infections (1, 2). They derive from *Streptomyces* species and are characterized by a macrocyclic lactone ring, which is either 14- [erythromycin (ERM), clarithromycin (CAM) and roxithromycin (RXM)], 15- [azithromycin (AZM)], or 16-membered (spiramycin, josamycin, midecamycin) (3). The antimicrobial activity of macrolides results from inhibition of bacterial protein synthesis through reversible binding to the peptide exit tunnel of ribosomes (4).

In addition to their antibiotic activity, macrolides have immunomodulatory properties, which were first described soon after their introduction in the 1950s (3, 5–7). The concept of using macrolides primarily for their immunomodulatory activities was introduced in the 1970s (8). The seminal study that distinguished between macrolides’ antimicrobial and their immunomodulatory effects was in adults with diffuse panbronchiolitis (DPB) in whom treatment with ERM dramatically improved survival independent of bacterial colonization (9). These results encouraged further research on the use of macrolides for the treatment of other chronic inflammatory conditions (10–14).

The mechanisms underlying the non-antimicrobial effects of macrolides are less well understood. Aside from ribosomal-mediated inhibition of pathogen virulence factor production, a number of other mechanisms have been proposed, including action on host immunity.

The objective of this review was to systematically summarize studies which investigated immunomodulatory properties of macrolides in humans and to describe the underlying mechanism and extent of their influence on the innate and adaptive immune system.

## Methods

**Figure 1:**
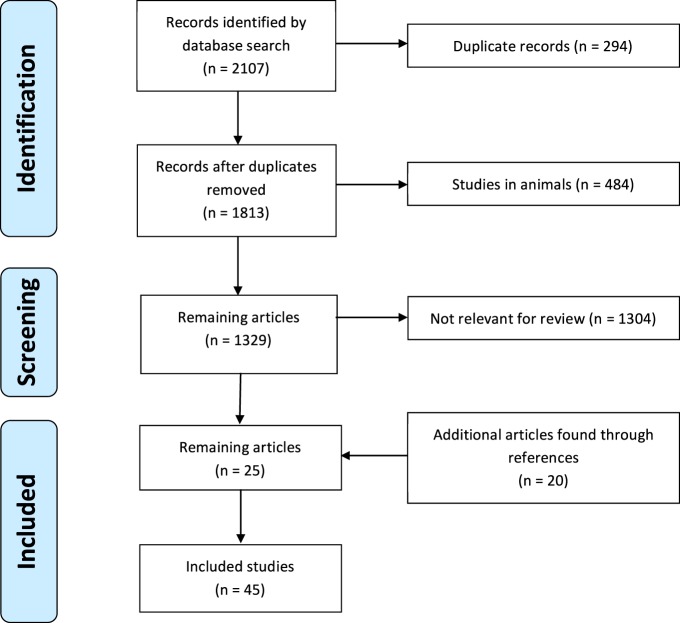
Selection of articles included in the review.

This review was done according to the preferred reporting items for systematic reviews and meta-analyses (PRISMA) (15). A literature review was done in December 2016 searching MEDLINE using the OVID interface from 1946 to 2016 using the search terms: (macrolide OR azithromycin OR clarithromycin OR erythromycin OR roxithromycin) AND (anti-inflammatory OR immunomodulatory OR immunolides) without any language limitations or limitation of study design (Figure 1). Only studies in humans, in which the participants received one of the four mentioned macrolides and which investigated immunological markers involved in inflammation were included. Studies reporting clinical endpoints only or studies in which macrolides were investigated for their antimicrobial activity were excluded. References were hand-searched for additional publications. Search results were independently screened by one reviewer, and checked by a second reviewer. Potentially eligible full-text articles were assessed according to our inclusion and exclusion criteria. The following variables were extracted from the included studies: year of study, country, study design, number of participants, age of participants, underlying disease, type, dose and duration of macrolide use, type of samples collected, and measured immune markers. Changes were classified as being significant when the *p*-value was ≤0.05.

## Review

### Characteristics of Included Studies

We identified 2,107 studies, of which 45 were included in the final analysis; 22 randomized, controlled trials, 16 prospective cohort studies, and 7 case–control studies (Figure 1). Studies originated from 17 countries (Japan *n* = 12, United States of America *n* = 6, China *n* = 4, Australia *n* = 4, United Kingdom *n* = 4, Turkey *n* = 2, Serbia *n* = 2, Croatia *n* = 2, and one each from Belgium, Canada, Greece, the Netherlands, Italy, South Korea, Russia, Sweden, and Switzerland) and included a total of 1,834 participants. Six studies, including 423 participants, were done in children and adolescents (<18 years of age). Details of all studies including a risk of bias analysis are summarise in Table 1 and Table 2.

**Table 1:** Macrolide-induced changes in immunological markers in 45 studies in humans categorized by disease (NS = not stated).

	Drug	Dose	Duration (weeks)	Patients (healthy) (*n*)	Age (years) (mean)	Sample	Measured immune markers	Study design	Reference
Blepharitis	AZM	1% topical drops	4	24 (8)	34–80 (54)	Conjunctival cells	Decrease in IL-1beta, IL-8, matrix metalloproteinase 9 (MMP-9)	CCS	Zhang et al. (16)
						Eyelid margins	Increase in TGF-beta		

Periodontitis	RXM	300 mg daily	0.7	47 (16)	28–65 (46)	Gingival cervicular fluid	Decrease in IL-1beta, TGF-beta, VEGF	RCT	Gong et al. (18)
	Placebo								

Nasal polyps	CAM	500 mg daily	8	40	25–73 (44)	Nasal secretions	Decrease in CCL-5 in allergic and non-allergic patients	PCS	Peric et al. (19)
							Decrease in IL-6 in allergic patients		
							Decrease in ECP in non-allergic patients		

	CAM	500 mg daily	8	40	25–73 (44)	Nasal secretions	Decrease in IL-8 in allergic and non-allergic patients	PCS	Peric et al. (20)
							Decrease in IL-1beta, IL-6 in allergic patients		
							Decrease in TNF-alpha in non-allergic patients		

	CAM	400 mg daily	12	20	28–84 (57)	Nasal secretions	Decrease in IL-8	PCS	Yamada et al. (21)

Rhinosinusitis	CAM	500 mg BID	2	25	19–70 (45)	Nasal mucosa cells	Decrease in macrophage count, eosinophil activity, neutrophil elastase, IL-6, IL-8, and TNF-alpha	PCS	MacLeod et al. (22)
	CAM	250 mg daily	12	30	25–63 (46)	Nasal secretions	Decrease in IL-8, ECP	PCS	Cervin et al. (23)
							Decrease in MPO, alpha2-macroglobulin (not significant)		
	CAM	250 mg daily	12	10	27–62 (48)*	Nasal mucosa cells	Decrease in TGF-beta, NF-kappaB (not significant)	PCS	Wallwork et al. (24)

	CAM	250 mg daily	52	17	18–67 (51)	Nasal mucosa cells	Increase in nasal nitric oxide (not significant)	PCS	Cervin et al. (59)
	ERM	250 mg BID							

	RXM	150 mg daily	1–46	12	16–73 (54)	Nasal secretions	Decrease in neutrophil count, IL-8	PCS	Suzuki et al. (25)

	RXM	150 mg daily	12	64	>18	Nasal secretions	Decrease in IL-8	RCT	Wallwork et al. (26)
	Placebo					Nasal mucosa cells			

Asthma Bronchiale	AZM	250 mg daily	12	71	18–70 (43)	Sputum	No change in eosinophil count, neutrophil count	RCT	Cameron et al. (27)
	Placebo								

	AZM	NS	12	40	22–52 (35)	Sputum	Decrease in IL-4, IL-5, IFN-gamma	RCT	He et al. (28)
	Placebo								

	AZM	10 mg/kg daily, 3 times a week	8	16	NS (13)	BAL	Decrease in neutrophil count	RCT	Piacentini et al. (29)
	Placebo								

	CAM	15 mg/kg BID (max 500 mg)	0.7	43	4–17 (9)	Nasopharyngeal secretions	Decrease in TNF-alpha, IL-1, IL-10	RCT	Fonseca-Aten et al. (30)
	Placebo								

	CAM	200 mg BID	8	17	26–49 (38)	Sputum	Decrease in eosinophil count, ECP	RCT	Amayasu et al. (31)
	Placebo					Blood	Decrease in eosinophil count, ECP		

	CAM	500 mg BID	6	86	NS (33)	BAL	Decrease in TNF-alpha, IL-5, IL-12	RCT	Kraft et al. (32)
	Placebo					Airway tissue	Decrease in TNF-alpha, IL-5, IL-12		

	CAM	500 mg BID	8	45	27–80 (58)	Sputum	Decrease in neutrophil count, neutrophil elastase, IL-8	RCT	Simpson et al. (33)
	Placebo						Decrease in MMP-9 (not significant)		

	CAM	500 mg BID	8	45	27–80 (60)	Sputum	Decrease in neutrophil count, neutrophil elastase, MMP-9, IL-8	RCT	Wang et al. (34)
	Placebo								

	RXM	150 mg BID	8	14	29–50 (40)	Sputum	Decrease in eosinophil count, ECP	RCT	Shoji et al. (35)
	Placebo					Blood	Decrease in eosinophil count, ECP		

	RXM	150 mg daily	12	20 (10)	NS (41)	PMNL	Decrease in neutrophil oxidative burst	PCS	Kamoi et al. (36)

Bronchiectasis	CAM	500 mg daily	12	22	32–78 (58)	Blood	Decrease in Th17-cells, IL-17	PCS	Fouka et al. (37)
	CAM	15 mg/kg daily	12	34	7–18 (13)	BAL	Decrease in total cell count, neutrophil count, IL-8	RCT	Yalcin et al. (38)
	Supportive treatment						Increase in macrophage count		
	RXM	150 mg daily	26	52	18–65 (48)	Sputum	Decrease in neutrophil count, neutrophil elastase, IL-8, MMP-9	RCT	Liu et al. (39)
	No treatment								

Chronic obstructive pulmonary disease	AZM Placebo	500 mg daily	0.4	24	35–70 (62)	Blood	Increase in neutrophil oxidative burst	RCT	Parnham et al. (40)
							Decrease in leukocyte count, thrombocyte count, IL-8, E-selectin, CRP, lactoferrin, serum amyloid A		
							No change in TNF-alpha, IL-6, GM-CSF		
						Sputum	No change in neutrophil count, eosinophil count		

	CAM	500 mg daily	12	67	NS (65)	Sputum	Decrease in neutrophil chemotaxis (not significant)	RCT	Banerjee et al. (41)
	Placebo						No change in total cell count, neutrophil count, IL-8, leukotriene B 4, TNF-alpha, neutrophil elastase		

	ERM Placebo	125 mg TDS	24	36	≥40 (69)	Sputum	Decrease in total cell count, neutrophil count, neutrophil elastase	RCT	He et al. (42)

Diffuse panbronchiolitis	ERM	250 mg BID	≥24	14	NS (46)	BAL	Decrease in lymphocyte count, IL-2, IFN-gamma Increase in CD4/CD8 ratio, IL-4, IL-5, IL-13	PCS	Park et al. (43)

	ERM	200 mg TDS	24–52	18 (5)	14–63 (39)	BAL	Decrease in total cell count, neutrophil count, neutrophil chemotaxis	CCS	Oda et al. (44)

	ERM	NS TDS	24–52	19	NS (42)	BAL	Decrease in total cell count, neutrophil count, neutrophil chemotaxis	CCS	Kadota et al. (45)
	ERM	200 mg TDS	8–68	22 (5)	18–70 (45)	BAL	Decrease in neutrophil count, neutrophil chemotaxis, IL-8	CCS	Katsuki et al. (46)
							No change in TNF-alpha		
	ERM	600 mg daily	12	12	16–75 (47)	BAL	Decrease in neutrophil count, neutrophil elastase	CCS	Ichikawa et al. (47)
	Amoxicillin								

	ERM	600 mg daily	4–104	43 (7)	(47)	BAL	Decrease in neutrophil count, IL-1beta, IL-8	CCS	Sakito et al. (48)
	RXM	150 mg daily							
	ERM	400 mg daily	12	12 (6)	NS	Blood	Decrease in neutrophil oxidative burst	CCS	Umeki (49)

Cystic fibrosis	AZM	NS	4	260	6–18 (NS)	Blood	Decrease in neutrophil count, MPO, high-sensitivity C reactive protein, serum amyloid A, calprotection	RCT	Ratjen et al. (50)

	AZM	250 mg daily (≤ 40 kg)	24	41	8–18 (NS)	Sputum	Decrease in IL-8, neutrophil elastase (not significant) (data only available from 17 patients)	RCT	Equi et al. (51)
		500 mg daily (> 40 kg)							

	CAM	7.5 mg/kg BID	12	18	3–15 (9)	BAL	Decrease in neutrophil count, neutrophil elastase (not significant)	RCT	Doğru et al. (52)
							Increase in macrophage count (not significant)		

	CAM	250 mg daily	52	27	6–17 (12)	Sputum	Decrease in IL-4, IL-8, TNF-alphaDecrease in INF-gamma (not significant)	PCS	Pukhalsky et al. (53)
						Blood	Decrease in IL-4, IL-8, TNF-alpha		

Lung transplantation	AZM	NS	12–24	30	36–61 (56)	BAL	Decrease in neutrophil count, IL-8, MMP-9	PCS	Verleden et al. (54)

Diabetic nephropathy	CAM	200 mg daily	12	16	NS (67)	Urine	Decrease CCL-2	RCT	Tone (60)
	Placebo					Blood			

Coronary atherosclerosis	CAM	500 mg daily	8	231	NS (65)	Blood	Decrease in CRP, IL-2, IL-6, IL-8, TNF-alpha (not significant)	RCT	Berg et al. (55)
	Placebo								

Healthy volunteers	AZM	500 mg daily	0.4	12	24–45 (29)	Blood	Increase in neutrophil oxidative burst, apoptosis of neutrophils	PCS	Culić et al. (56)
							Increase in TNF-alpha (not significant)		
							Decrease in IL-1beta, IL-6, IL-8, myeloperoxidase, IL-17, soluble vascular cell adhesion molecule-1		
							Decrease in E-selectin, lactoferrin (not significant)		
							No change in leukocyte count, thrombocyte count, neutrophil elastase, beta2-microglobulin, INF-gamma, GM-CSF		

	AZM	500 mg on day 1, then 250 mg	0.7	12	23–47 (30)	Sputum	No change in total cell count, neutrophil count, IL-6, IL-8 after ozone exposure during exercise	RCT	Criqui et al. (57)
	Placebo								

	AZM	500 mg daily	0.4	19	18–40 (25)	BAL	No change in TNF-alpha, IL-1beta, IL-6, superoxide generation by alveolar macrophages	PCS	Aubert et al. (58)
						Blood			

	AZM	500 mg daily first day, then 250 mg daily	0.4	10	NS (30)	Gingival cervicular fluid	Decrease in IL-1beta, IL-8, TNF-alpha, VEGF	PCS	Ho et al. (17)

**Table 2:** Risk of bias summary of the randomized controlled trials and case–control studies included in the review (NS = not stated).

Reference	Publication year	Selectionbias	Performancebias	Detectionbias	Attritionbias	Reportingbias
**Randomized controlled trials**						

**Periodontitis**						

Gong et al. (18)	2013	−	−	−	−	−

**Rhinosinusitis**						

Wallwork et al. (26)	2006	−	−	−	−	−

**Asthma bronchiale**						

Cameron et al. (27)	2013	NS	−	−	+	+
He et al. (28)	2009	+	NS	NS	NS	+
Piacentini et al. (29)	2007	−	−	−	+	+
Fonseca-Aten et al. (30)	2006	+	−	−	−	+
Amayasu et al. (31)	2000	+	−	−	−	−
Kraft et al. (32)	2002	+	−	−	−	+
Simpson et al. (33)	2008	−	−	−	−	+
Wang et al. (34)	2012	+	NS	NS	NS	−
Shoji et al. (35)	1999	+	−	−	−	+

**Bronchiectasis**						

Yalcin et al. (38)	2006	+	NS	NS	−	+
Liu et al. (39)	2014	+	+	+	−	+

**Chronic obstructive pulmonary disease**						
Parnham et al. (40)	2005	+	−	−	−	−
Banerjee et al. (41)	2004	+	−	−	−	−
He et al. (42)	2010	+	−	−	−	−

**Cystic fibrosis**						

Ratjen et al. (50)	2012	−	−	−	−	−
Equi et al. (51)	2002	+	−	−	−	+
Doğru et al. (52)	2009	+	−	−	−	−

**Diabetic nephropathy**						
Tone et al. (60)	2011	+	+	+	−	−

**Coronary atherosclerosis**						
Berg et al. (55)	2003	+	−	−	−	−

**Healthy volunteers**						
Criqui et al. (57)	2000	+	−	−	−	−

**Case–control studies**						

**Blepharitis**						
Zhang et al. (16)	2015	+	+	+	−	−

**Diffuse panbronchiolitis**						
Oda et al. (44)	1994	+	+	+	−	−
Kadota et al. (45)	1993	+	+	+	−	−
Katsuki et al. (46)	1996	+	+	+	−	−
Ichikawa et al. (47)	1992	+	+	+	+	−
Sakito et al. (48)	1996	+	+	+	−	−
Umeki (49)	1993	−	+	+	−	−

### Immunological Markers Analyzed

**Figure 2:**
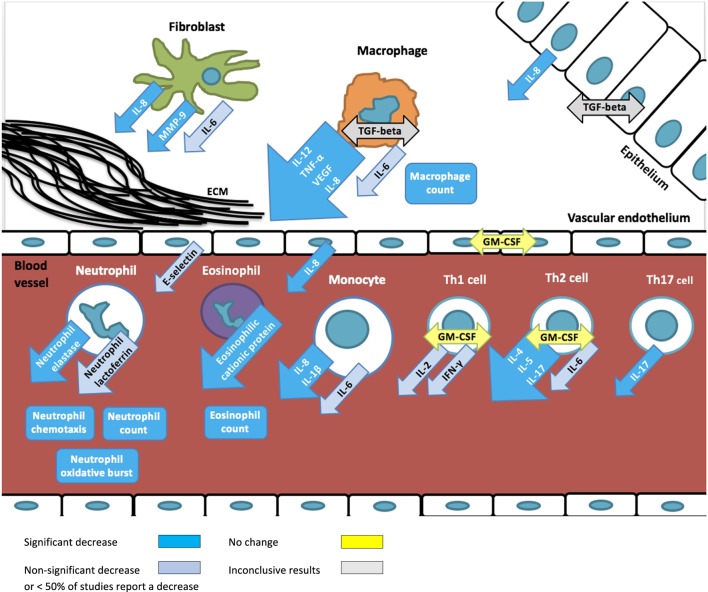
Overview of immunomodulatory effects of macrolides based on studies summarized in Table 1. Arrows depict excreted proteins, boxes depict cell counts or functions.

A total of 47 different immunological markers were investigated. On average, four markers were investigated per study resulting in a total of 186 measurements (Table 3; Figure 2). The immunological markers were classified into groups: cell counts (*n* = 9 markers/41 total measurements), neutrophil function (*n* = 6/25), eosinophil function (*n* = 2/7), macrophage function (*n* = 1/1), cytokine concentrations (*n* = 16/81), inflammatory proteins (*n* = 6/8), cell adhesion molecules (*n* = 2/3), molecules involved in inflammatory signaling pathway (*n* = 1/1), and other markers (*n* = 5/5, alpha-2-macroglobulin, beta-2-microglobulin, high-sensitivity C reactive protein, calprotectin, nasal nitric oxide).

**Table 3:** Macrolide-induced changes in immunological markers based on 43 studies in humans.

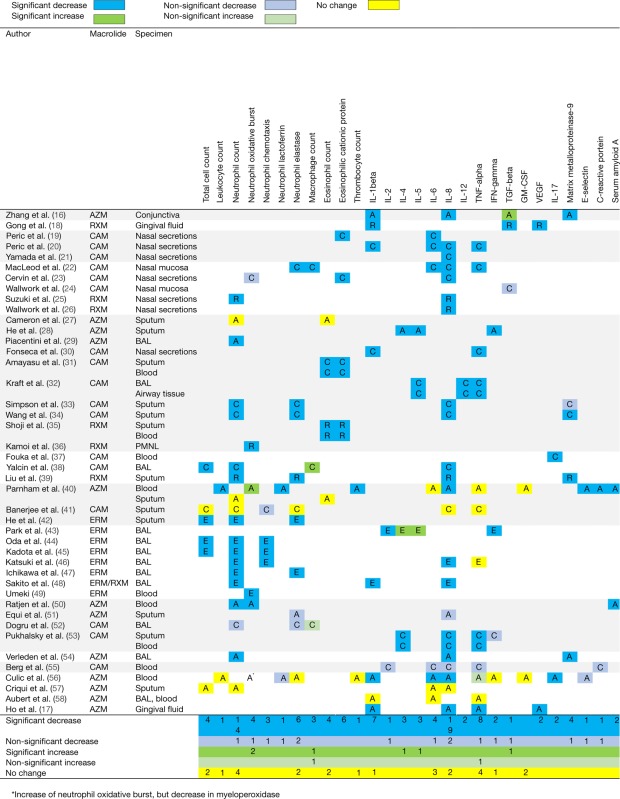

Overall, a decrease in immunological markers, number, or function was more frequently observed than an increase (139 measurements vs 19). No change of immunological markers reported in 11 immunological markers (28 measurements) in 7 studies. The most frequently reported macrolide-induced changes were a decrease in interleukin (IL)-8 concentration (*n* = 21), neutrophil count (*n* = 15), tumor necrosis factor-alpha (TNF-alpha) (*n* = 9), neutrophil elastase (*n* = 8), IL-1beta (*n* = 7), eosinophilic cationic protein (ECP, *n* = 6), IL-6 (*n* = 5), matrix metalloproteinase 9 (MMP-9) (*n* = 5), and oxidative burst activity (*n* = 5).

Immunomodulatory effects were investigated for four types of macrolides, including CAM (*n* = 73), AZM (*n* = 69), ERM (*n* = 27), and RXM (*n* = 17). AZM was more frequently associated with no influence on the immunological markers investigated (21/69) compared to any of the other macrolides (Table 4).

**Table 4:** Number of measurements and changes in immunological markers for each macrolide.

	Decrease/non-significant decrease	Increase/non-significant increase	No change	Total
AZM	33/4	10/1	21	69
CAM	52/12	1/2	6	73
ERM	21/0	4/1	1	27
RXM	17/0	0/0	0	17
Total	123/16	15/4	28	186

### Immunomodulatory Properties in Different Diseases

In the following, the immunomodulatory properties of macrolides are summarized and categorized by the disease in which they were investigated (Table 1).

### Blepharitis

Blepharitis is a common chronic inflammation of the eye lid leading to dry, itchy, and erythematous eyes. Anterior blepharitis is often associated with bacterial infections, while posterior blepharitis is linked to dysfunction of Meibomian glands. Many studies report clinical improvement in patients with blepharitis treated with topical AZM, due to a decrease in secretions and plugging of the Meibomian glands but did not investigate the underlying immunological mechanisms. The one study which did investigate immunological markes shows that concentrations of IL-1beta, IL-8, and MMP-9 in conjunctival cells of patients with blepharitis are higher than in healthy controls (16). Concentrations of these cytokines decrease with local AZM treatment, but return to pre-treatment levels after discontinuation (16).

### Periodontitis

Periodontitis is an inflammatory process of the gums with a complex pathogenesis including microorganisms as well as neutrophils, macrophages and fibroblasts. One key immunological mechanism underlying the pathogensis of periodontitis has been described as a TNF-alpha-induced increase in vascular endothelial growth factor (VEGF) leading to an aberrant angiogenesis (61). Both AZM and RXM decreased TNF-alpha and VEGF concentrations as well as other cytokines including IL-1beta, IL-8, and transforming growth factor beta (TGF-beta) in gingival crevicular fluid (17, 18). Since oral bacteria play an important role in periodontitis, however, some of the some of the benefits of macrolides may be attributable to antimicrobial rather than to immunomodulatory effects.

### Chronic Rhinosinusitis and Nasal Polyposis

Chronic rhinosinusitis (CRS) with nasal polyps is characterized by a T helper (Th) 2 cells-dominated inflammation with upregulation of IL-4, IL-5, and IL-13 and an increase in eosinophil count, ECP, and immunoglobulin E. CRS without nasal polyps is characterized by Th1-dominated inflammation with upregulation of IL-2, TGF-beta, and IFN-gamma. Studies in patients with CRS treated with CAM and RXM show a significant reduction in macrophage, neutrophil, and eosinophil counts and concentrations of neutrophil elastase, ECP, CC-chemokine ligand-5 (CCL-5), IL-1beta, IL-6, IL-8, interferon (IFN)-gamma, TNF-alpha, myeloperoxidase (MPO), and alpha-macroglobulin in nasal secretions (19–23, 25, 26, 62). One of the postulated mechanisms by which macrolides inhibit the development of nasal polyps is through their anti-oxidative effects inhibiting the TGF-beta-induced production of reactive oxygen species (24). However, the immunomodulatory mechanisms differ in allergic and non-allergic nasal polyposis patients. While CAM reduces IL-6 and CCL-5 in all patients, it reduces IL-1beta and IL-6 only in patients with allergic CRS and TNF-alpha and ECP only in patients with non-allergic CRS (19, 20).

### Asthma

Asthma is characterized by chronic airway inflammation, reversible airway obstruction, and airway hyper-responsiveness. In eosinophilic asthma, eosinophils, mast cells, and Th2-mediated inflammation play an important role. Concentrations of IL-4, IL-5, IL-6, IL-9, IL-10, IL-13, vascular cell adhesion molecule-1, CC chemokines, and granulocyte macrophage colony-stimulating factor (GM-CSF) are elevated. In severe asthma, in addition to eosinophils, increased neutrophils and IL-8 concentrations are found in airways. In patients with asthma, AZM, CAM, and RXM decrease eosinophil and neutrophil counts, inhibit neutrophil migration and oxidative burst activity in phagocytes, decrease concentrations of neutrophil elastase, ECP, IL-1, IL-4, IL-5, IL-8, IL-10, IL-12, MMP-9, TNF-alpha, and INF-gamma in nasopharyngeal secretions, sputum, or bronchoalveolar lavage (BAL) samples (27–30, 32–36). In addition, CAM and RXM also decrease the eosinophil counts and concentrations of ECP in blood and inhibit oxidative burst activity in phagocytes (31, 35, 36).

### Bronchiectasis

Bronchiectasis is characterized by permanent enlargement of bronchi and cytokines play an important role in the pathogenesis. In BAL samples of patients with bronchiectasis, elevated concentrations of IL-1beta and IL-8, as well as Th17-cytokines (IL-17A and IL-23), are found. In this setting, CAM and RXM lead to a decrease in total cell and neutrophil counts, concentrations of neutrophil elastase, IL-8, and MMP-9 in sputum or BAL of patients with bronchiectasis (38, 39). Interestingly, in BAL samples, these drugs significantly increase macrophage counts (38). Furthermore, macrolides lead to a decrease in peripheral blood Th17 cells and IL-17 concentrations (37).

### Chronic Obstructive Pulmonary Disease

Chronic obstructive pulmonary disease (COPD) is characterized by chronic inflammation of lung parenchyma and peripheral airways with an increase in alveolar macrophages, neutrophils, T cells (predominantly Th1-, and Th17- cells), and innate lymphoid cells. These cells, as well as structural cells, such as epithelial cells, endothelial cells, and fibroblasts, secrete a variety of pro-inflammatory cytokines. Although most patients with COPD have a predominantly neutrophilic inflammation, some also have elevated eosinophil counts in sputum. Oxidative stress plays a key role in COPD, and can result in activation of the pro-inflammatory transcription factor nuclear factor (NF)-kappaB. Moreover, COPD is associated with increased apoptosis and defective phagocytosis in the airways. In patients with COPD, IL-1beta, IL-4, IL-8, and TNF-alpha concentrations in blood are elevated, while IL-10 concentrations are lower compared to healthy adults. In patients with COPD, AZM leads to a decrease in white blood cell and platelet counts and concentrations of CRP, IL-8, E-selectin, and lactoferrin in blood (40). By contrast, macrolides increase neutrophil oxidative burst and neutrophil glutathione peroxidase activity in blood (40). In the sputum of COPD patients, CAM and ERM lead to a significant decrease in total cell and neutrophil count and inhibit neutrophil chemotaxis and decrease concentrations of neutrophil elastase (41, 42).

### Diffuse Panbronchiolitis

Diffuse panbronchiolitis (DPB) is a chronic distal airway inflammation characterized by diffuse micronodular pulmonary lesions mostly consisting of neutrophils. Neutrophils and epithelial cells produce IL-8, which is an important chemotactic factor to attract more neutrophils. The neutrophil count in BAL samples of patients with DPB correlates to the concentrations of IL-1beta and IL-8 (48). ERM reduces IL-1beta concentrations in BAL samples of patients with DPB which leads to a subsequent reduction of IL-8 concentrations and a decrease in neutrophil count and neutrophil chemotactic activity (44–48, 63). Furthermore, ERM treatment also results in a decrease in lymphocyte count, IL-2, interferon-gamma, and to increase in CD4/CD8 ratio, IL-4, IL-5, IL-13 in BAL samples of patients with DPB (43).

### Cystic Fibrosis

In patients with cystic fibrosis (CF), chronic airway inflammation results from cytokines secreted by epithelial and immune cells, which leads to neutrophil influx into airways. The release of neutrophil proteases, including neutrophil elastase, contributes to the development of bronchiectasis. Sustained inflammation is mainly due to an increase in the transcription of NF-kappaB activity, which leads to an increase in IL-8 production. These immunological mechanisms are influenced by AZM and CAM, which in CF-patients lead to a decrease in neutrophil count, concentrations of neutrophil elastase, IL-4, IL-8, TNF-alpha, and INF-gamma, and to an increase in numbers of macrophages in BAL samples or in sputum (51–53). In CF-patients macrolides also lead to a decrease in neutrophil count, concentrations of IL-4, IL-8, TNF-alpha, MPO, high-sensitivity C reactive protein, serum amyloid A, and calprotectin in blood (50, 53).

## Discussion

Macrolides are important therapeutic options in the treatment of many chronic inflammatory diseases because of their immunomodulatory effects. To understand the mechanisms underlying these effects, we reviewed all human studies that analyzed the influence of macrolides on immunological markers. The non-antimicrobial effects of macrolides are extensive and range from changes in cell counts and function, up- and downregulation of cytokine production to expression of adhesion molecules.

The most frequently and consistently reported immunomodulatory effect of macrolides is a reduced neutrophilic inflammation. Reduced numbers of neutrophils and inhibition of neutrophilic function lead to lower concentrations of neutrophil elastase and IL-8, and ultimately to a decrease in tissue injury. Furthermore, macrolides also reduce IL-1beta concentrations, another key mediator of the inflammatory response that is most abundantly produced by monocytes and macrophages. Evidence from animal and *in vitro* studies show that the inhibition of the key pro-inflammatory cytokines IL-8 and IL-1beta results from macrolides’ ability to alter intracellular signaling, particularly through the inhibition of NF-kappaB activation and expression of activator protein-1 (64–66). Notably, this effect has been observed in the absence of an infectious agent.

On the basis of these observed *in vitro* immunological effects of macrolides, patients with diseases mediated by neutrophilic inflammation such as periodontitis, severe asthma, DPB, bronchiectasis, COPD, and CF should benefit from treatment with this class of antibiotics. Indeed, clinically beneficial effects have been shown in randomized controlled studies in patients with COPD and CF with improved symptom scores, respiratory function and decreased frequency of exacerbations (67–69). For DPB, bronchiectasis and asthma, however, there is an absence of randomized controlled studies showing clinical beneficial effects of macrolides (70–72).

Macrolides are more commonly and consistently reported to inhibit neutrophilic than eosinophilic function. This is consistent with clinical studies that show patients with eosinophil-driven chronic inflammatory diseases associated with increased IgE (such as CRS or atopic asthma) have significantly lower improvement rates with macrolide treatment than those with normal serum IgE (26, 62, 73). Although the effect of macrolides on eosinophils has been less commonly investigated, a few studies report decreased eosinophil counts, and concentration of ECP (a ribonuclease secreted by eosinophils responsible for local cytotoxic effect). This suggests that there may be a role for the use of macrolides in allergic chronic inflammatory diseases (43, 74, 75). The possible influence of macrolides on eosinophilic inflammation is further supported by the finding that Th2 cytokines, such as IL-4 and IL-5, are more frequently reduced than Th1 cytokines, such as IL-2 and INF-gamma (19, 20, 22, 32, 42, 43, 53, 55, 56). The stronger effect of macrolides on Th2 compared with Th1 responses is further supported by evidence from animal and *in vitro* studies (74, 75). However, some of the anti-inflammatory effects might also be explained through their antibiotic effect on (undiagnosed) pathogens which trigger and sustain inflammation.

It is likely that immunomodulatory effects vary between different macrolides. Although some studies included more than one macrolide, none of the human studies directly compared different macrolides. Interestingly, AZM was less frequently associated with changes in measured immunological markers compared to the other macrolides. However, most of these studies were either in healthy volunteers or AZM was administered for only a few days (56–58, 76). By contrast, clinical studies in patients with CF suggest that AZM, but not CAM, leads to an improvement in respiratory function and reduction in pulmonary exacerbations (69, 77). *In vitro* studies comparing the immunomodulatory effects of different macrolides suggest that CAM has less immunomodulatory activity compared to other macrolides. For example, RXM, but not CAM or ERM, was shown to decreased chemotaxis of Th1 and Th2 cells (78). Similarly, CAM had a significantly weaker effect on reducing IL-6 production by human macrophages than ERM (79). Furthermore, another study showed that AZM, but not CAM or RXM, inhibits IL-1alpha and IL-1beta production (80).

Immunomodulatory effects of macrolides have been described with the recommended dose for antimicrobial treatment. Macrolides have excellent tissue penetration compared to other classes of antibiotics resulting in tissue concentrations generally exceeding serum concentrations (except for RXM). For the immunomodulatory effects macrolides’ ability to accumulate in neutrophils and macrophages is particularly important. Concentrations in macrophages have been shown to be 400- to 800-fold higher compared to serum for CAM and AZM and 5- to 100-fold higher in tissue compared to serum for ERM, CAM and AZM (81–85). This drug accumulation in immune cells may result in immunomodulatory effects occurring at lower doses and lasting longer compared to the antimicrobial effects. The relationship between macrolide dose and immunomodulatory effect is, therefore, an interesting avenue for future research.

The main limitation of this review is the heterogeneity of study populations, underlying diseases, type of macrolide and methods used to assess the immunomodulatory effect. A further limitation is selection and reporting bias and based on study types other biases including carry-over effect in cross-over trials and recall bias in case–control studies.

In summary, there is substantial evidence that macrolides exhibit immunomodulatory effects through inhibition of neutrophilic inflammation and macrophage activation. However, there is considerable heterogeneity between studies and in the immunological markers measured. Further studies will help delineate the exact mechanisms underlying the immunomodulatory properties of macrolides and the relative activity of different macrolides. This will enable the optimal use of this class of antibiotics in the treatment of chronic inflammatory diseases.

## Author Contributions

PZ and NC designed the study. PZ drafted the initial manuscript and approved the final manuscript as submitted. PZ, VZ, and NR did the risk of bias analysis. VZ, NC, and NR critically reviewed and revised the manuscript, and approved the final manuscript as submitted.

## Conflict of Interest Statement

The authors declare that the research was conducted in the absence of any commercial or financial relationships that could be construed as a potential conflict of interest.
